# Tensor clustering with algebraic constraints gives interpretable groups of crosstalk mechanisms in breast cancer

**DOI:** 10.1098/rsif.2018.0661

**Published:** 2019-02-06

**Authors:** Anna Seigal, Mariano Beguerisse-Díaz, Birgit Schoeberl, Mario Niepel, Heather A. Harrington

**Affiliations:** 1 Department of Mathematics, University of California, Berkeley, CA 94702, USA; 2 Mathematical Institute, University of Oxford, Oxford OX2 6GG, UK; 3 Novartis Institutes for BioMedical Research, Cambridge, MA 02139, USA; 4 Ribon Therapeutics, Lexington, MA 02421, USA

**Keywords:** algebra, tensors, data clustering, signalling networks, systems biology, model selection and parameter inference

## Abstract

We introduce a tensor-based clustering method to extract sparse, low-dimensional structure from high-dimensional, multi-indexed datasets. This framework is designed to enable detection of clusters of data in the presence of structural requirements which we encode as algebraic constraints in a linear program. Our clustering method is general and can be tailored to a variety of applications in science and industry. We illustrate our method on a collection of experiments measuring the response of genetically diverse breast cancer cell lines to an array of ligands. Each experiment consists of a cell line–ligand combination, and contains time-course measurements of the early signalling kinases MAPK and AKT at two different ligand dose levels. By imposing appropriate structural constraints and respecting the multi-indexed structure of the data, the analysis of clusters can be optimized for biological interpretation and therapeutic understanding. We then perform a systematic, large-scale exploration of mechanistic models of MAPK–AKT crosstalk for each cluster. This analysis allows us to quantify the heterogeneity of breast cancer cell subtypes, and leads to hypotheses about the signalling mechanisms that mediate the response of the cell lines to ligands.

## 1. Introduction

Muti-dimensional datasets are prevalent across the sciences; their ubiquity and importance will only continue to grow [1–4]. The ever-increasing sophistication of datasets requires the development of methods that preserve multi-dimensional structures and exploit them, while maintaining interpretability of results. In addition, clustering biological data is far from a straightforward task. There are multiple challenges, including choosing an appropriate method for the data [5], handling high-dimensional data [6,7] and, importantly, the consideration of the biological context of the problem, which must be done almost on a case-by-case basis [8].

Among the wide variety of clustering methods, constrained clustering is an active field of research [9–13]. The most common approaches incorporate pairwise *must-link* and *cannot-link* constraints to indicate whether two items must or must not be in the same cluster [14,15]. Other methods set constraints on what the possible clusters can be, rather than constraining the elements in a cluster [16]. In these cases, there is a large pool of candidate clusters from which those that meet selection criteria can be chosen.

In this work, we introduce a versatile data clustering framework based on tensors and algebra to analyse high-dimensional datasets. One key feature of our method is that it can incorporate general, application-specific constraints on the composition of clusters, and is guaranteed to find optimal partitions. The flexibility of the method allows it to be used directly on a dataset (i.e. as a standalone clustering tool), or in combination with other clustering methods.

**Figure 1.**
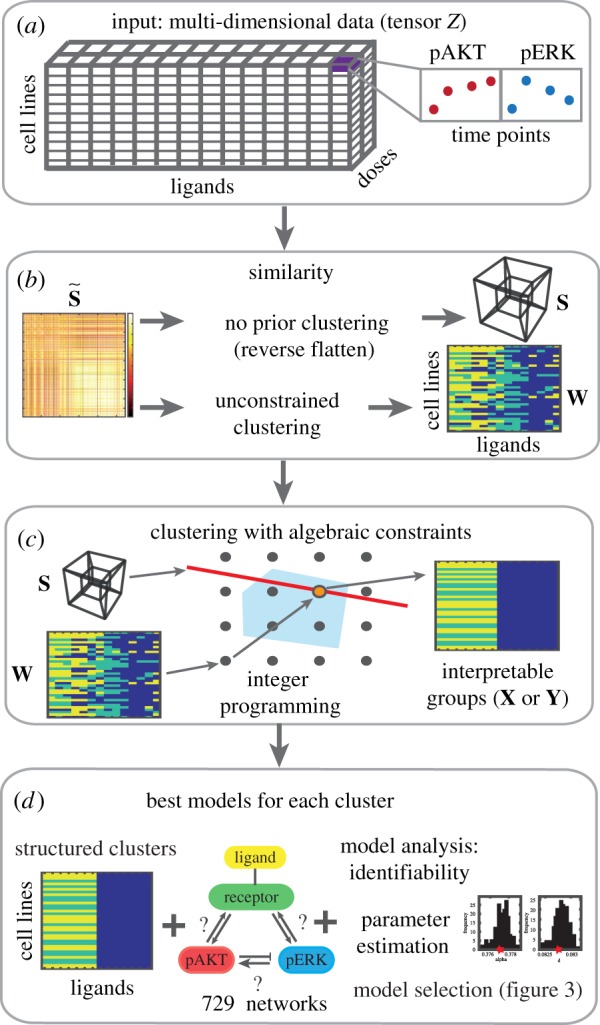
Schematic of the constrained tensor clustering method and model identification. (*a*) The complete set of experiments can be represented by the multi-indexed tensor **Z**; see §3. (*b*) The similarity scores between experiments (each cell line/ligand combination) can be stored in a similarity matrix $\tilde{S}$ that can be used to construct a similarity tensor **S**, or to find a preliminary clustering of the data **W** that may not comply with the constraints. (*c*) Structured clustering via integer programming. The starting point can be either the similarity tensor **S** or the pre-existing clustering **W**. The possible clusterings are represented by points on the grid. The red line is the value of the objective function (equations (4.2) and (4.3)). The best integer value (orange point) is found inside the convex feasible region (blue). (*d*) A large-scale search for mechanistic models for each cluster involves parametrizing, and ranking the best ODE models for each cluster. (Online version in colour.)

We showcase our clustering framework on an extensive set of time-course measurements of the activation levels of the mitogen-activated protein kinase (MAPK) and phosphoinositide 3-kinase (PI3K) pathways that are involved in cellular decisions and fates [17–20] and are known to dysfunction in cancer [21–25]. The key signalling proteins and subtype responses in breast cancer cells are known; however, among genetically diverse cell lines the specific dysfunction mechanisms vary and are not well understood [26–28]. We examine a set of experimental data [26] containing the response of 36 breast cancer cell lines after exposure to 14 ligands (growth factors/signalling molecules). Each experiment measures the temporal phosphorylation response of one cell line to one ligand. Because the dataset is *complete* (i.e. there is a measurement for every combination of times, proteins, cell lines, ligands and doses), we can represent it as a tensor in five dimensions (figure 1*a*).

**Figure 2.**
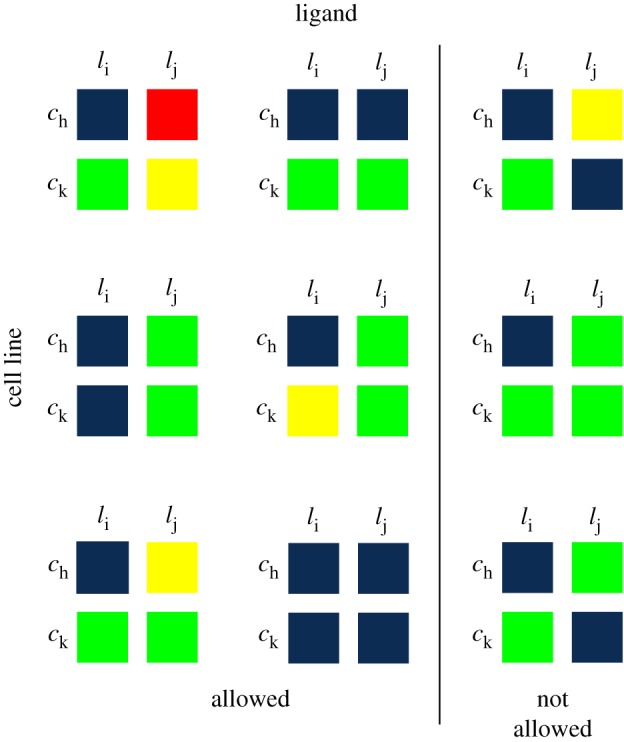
Examples of cluster shapes that are allowed and not allowed in our analysis of breast cancer data. The clusters in the first two columns are all rectangular, and thus allowed under our interpretability framework. The third column contains examples of non-rectangular clusters that are not allowed in our framework. Note that *j* is not necessarily equal to *i* + 1, and *k* is not necessarily *h* + 1. (Online version in colour.)

We find clusters of experiments subject to *interpretability constraints* (figure 1*b*,*c*). Our objective is to attribute differences between clusters to differences in the underlying signalling mechanisms, so the composition of the clusters must facilitate mechanistic interpretation. For example, the cell lines in a cluster could share a mutation, and the ligands are those whose effect is altered by the mutation. For this reason, we constrain the clusters to be rectangular, i.e. to match a subset of cell lines with a subset of ligands (figure 2). The constraints help to rule out similarities between experimental measurements that are incompatible with a mechanistic interpretation. The interpretability constraints take the form of algebraic inequalities.

We introduce a new notion of tensor similarity, which we employ to find optimal clusterings. The global optimality of the partitions is guaranteed by leveraging results from integer programming. One of the strengths of this approach is that it can incorporate a pre-existing non-rectangular partition obtained with other methods (e.g. conventional agglomerative clustering, *k*-means, spectral methods, community detection on graphs) and find the nearest optimal rectangular clustering. The distance between partitions is given by the number of experiments whose clustering assignment changes. Hence this method can be used in conjunction with any other state-of-the-art method and preserve the features that are compatible with the constraints. Moreover, using the method from an initial partition is computationally advantageous. The partition into clusters can be visualized by colour coding the grid of experiments according to their cluster assignment (figure 1*c*). Each box on the grid represents the cluster assignment of an entire vector (or even a tensor) of data.

Once we obtain an optimal partition of the data, the second stage of our analysis is to search for mechanisms that can explain the behaviour of the experiments in each cluster. We perform a systematic search for nonlinear ordinary differential equation (ODE) models that reproduce the key dynamical features of the time series in each cluster (figure 1*d*). To this end, we construct, parametrize and rank models for each cluster from a pool of 729 candidate models.

## 2. Tensors and algebra

### 2.1. Data tensor

We represent a multi-indexed dataset (e.g. the complete dataset in figure 1*a*) as a tensor **Z** of order *h* in the real numbers with size *n*_1_ × · · · × *n*_*h*_ (i.e. $Z\in R^{n_{1}\times \cdots \times n_{h}}$, where $n_{i}\in N$ and *i* = 1, …, *h*). When the dataset is complete, every entry of the tensor is filled with a number. A full treatment of tensors is available in [1] and references therein. We introduce here the tensor theory required for our analysis.

### 2.2. Similarity tensors

In a similarity matrix the entry (*i*, *j*) records the pairwise similarity of the two items labelled by unidimensional indices *i* and *j*. We now introduce the high-dimensional generalization of a similarity matrix, which extends this to multi-indexed data. Suppose we want to compute the similarity of the data indexed by **i** = (*i*_1_, *i*_2_) and indexed by **j** = (*j*_1_, *j*_2_),

2.1 $$s_{i,j}=\text{sim}(Z(i_{1},i_{2},:,\ldots ,:),\;Z(j_{1},j_{2},:,\ldots ,:)),$$

where *i*_1_, *j*_1_ ∈ {1, …, *n*_1_} and *i*_2_, *j*_2_ ∈ {1, …, *n*_2_}. The similarity function $\text{sim}:\;R^{n_{3}\times \cdots \times n_{h}}\times R^{n_{3}\times \cdots \times n_{h}}\to R$ computes the similarity between the data indexed by **i** and **j** (e.g. correlation or cosine similarity). In general, for data indexed by the first *d* dimensions, we have the multi-indices **i** = (*i*_1_, …, *i*_*d*_) and **j** = (*j*_1_, …, *j*_*d*_). The dimensions of **Z** can be re-ordered as needed. We can construct a *similarity tensor* **S** of order 2*d*. The shape of **S** is determined by the chosen dimensions of the data: $S\in R^{n_{1}\times \cdots \times n_{d}\times n_{1}\cdots \times n_{d}}$. The similarity tensor and the similarity matrix are related by *flattening* the tensor as follows. The original data tensor **Z** can be flattened (re-shaped) into a data matrix $\tilde{Z}\in R^{N_{1}\times N_{2}}$, where $N_{1}=\prod_{r=1}^{d}n_{r}$ and $N_{2}=\prod_{r=d+1}^{h}n_{r}$. Each row of $\tilde{Z}$ is an *N*_2_-dimensional vector that corresponds to multi-index **i**, and the length *N*_2_ is the product of the dimensions of **Z** that are *not* included in **i**.

The similarity matrix between the rows of $\tilde{Z}$ is $\tilde{S}\in R^{N_{1}\times N_{1}}$, which is obtained by flattening the similarity tensor, **S**. We summarize this relationship in the following diagram:


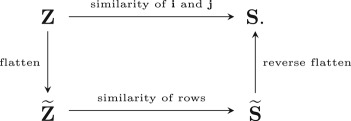


To compute the similarity tensor **S**, we can simply flatten the data tensor **Z** into $\tilde{Z}$, construct a similarity matrix $\tilde{S}$, and then reverse flatten it into the desired **S**. Note that **Z** and **S** have the same number of entries as $\tilde{Z}$ and $\tilde{S}$, respectively.

**Example.**

Let $Z\in R^{10\times 5\times 3}$ be a tensor of order 3. If **i** = (*i*_1_, *i*_2_) is the multi-index, then *d* = 2, $N_{1}=10\cdot 5=50$ and *N*_2_ = 3. The (order 4) similarity tensor **S** has size 10 × 5 × 10 × 5. The similarity matrix $\tilde{S}$ has size 50 × 50. The flattened data matrix $\tilde{Z}$ has size 50 × 3.

### 2.3. Algebraic interpretability condition

When clustering a set of data points we typically seek a partition such that the points within a cluster are more similar (or close) to each other than to the rest of the data [5]. In the simplest cases, there are few restrictions on the clusters other than that the similarity or distance be reflected in the cluster assignments. In certain cases, imposing restrictions on the clusters can be desirable or even required [11]. Here we pursue *structured clustering*; that is, we impose restrictions on the shape of the clusters in the tensor. In this application, we seek clusters with a rectangular shape, which allows us to interpret clusters in terms of data-generating mechanisms (i.e. grouping cell lines/ligand combinations to ensure mechanistic interpretation). We describe the biological motivation for these constraints in the results section (§5.1) and the mathematical details of the method here.

A hard partition of a dataset represented as a tensor **Z** of size *n*_1_ × · · · × *n*_*h*_ into *m* clusters can be encoded in two ways.

(1) An (*n*_1_ × · · · × *n*_*d*_) × (*n*_1_ × · · · × *n*_*d*_) tensor **X** in which the data have multi-indices **i** = (*i*_1_, …, *i*_*d*_) and **j** = (*j*_1_, …, *j*_*d*_), and:
  2.2 $$x_{ij}=\left\{\begin{array}{ll} 0 & \text{if}\;i\;\text{and}\;j\;\text{belong to the same cluster},\\ 1 & \text{otherwise.}\; \end{array}\right.$$
  The tensor **X** can be seen as a Boolean approximation of the distances between pairs of data points: *x*_**ij**_ = 0 if **i** and **j** are ‘close’ (in the same cluster), and *x*_**ij**_ = 1 if they are ‘far’ (in different clusters). To ensure that **X** encodes a valid clustering of the data, the three conditions of an equivalence relation must be met. These conditions are given by the following algebraic equations and inequality:
  2.3 $$\left.\begin{array}{rl} \mathrm{reflexivity}: & \;x_{ii}=0,\\ \mathrm{symmetry}: & \;x_{ij}=x_{ji}\\ \mathrm{and}\;\mathrm{transitivity}: & \;0\leq -x_{ik}+x_{ij}+x_{jk}\leq 2. \end{array}\right\}$$
(2) In an *n*_1_ × · · · × *n*_*d*_ × *m* tensor **Y**, where
  2.4 $$y_{ik}=\left\{\begin{array}{ll} 1 & \text{if the data indexed by}\;i\;\text{belongs to cluster}\;k,\\ 0 & \text{otherwise.}\; \end{array}\right.$$
  We require that $\sum_{k=1}^{m}y_{ik}=1$ to ensure that each data item has been assigned to exactly one cluster.

The tensors **X** and **Y** are related by the following equation:

$$1-x_{i,j}=\sum_{k=1}^{m}y_{i,k}y_{j,k}.$$

### 2.4. Integer optimization

The structural or interpretability conditions we have imposed on the clusters take the form of linear constraints. These constraints, along with the fact that the tensors are Boolean, allow us to find optimal tensors **X** and **Y** by solving an integer linear program [29,30]. Specifically, we use the branch and cut algorithm [31] as we describe in the Structured clustering section (§4) below.

## 3. Data

We examine an extensive experimental dataset detailing the temporal phosphorylation response of signalling molecules in genetically diverse breast cancer cell lines in response to different growth factors [26]. This dataset is complete and can be represented by a tensor **Z** of order 5 whose dimensions correspond to 36 cell lines, 14 ligands, two doses, three time points and two proteins (pERK, pAKT) (for more details, see the electronic supplementary material, appendix). In this work, each experiment is a set of measurements (for all time points, doses and proteins) for each cell line/ligand combination (36⋅14=504 experiments). Our goal is to find sets of experiments with a similar response; consequently, the data structures we require are the following:

$$\begin{array}{ll} & Z\in R^{36\times 14\times 2\times 3\times 2},\;\text{( data tensor)}\\ & \tilde{Z}\in R^{504\times 12},\;\;\;\text{( flattened data tensor) }\\ & S\in R^{36\times 14\times 36\times 14}\;\;\;\text{( similarity tensor)}\\ \mathrm{and}\;\; & \tilde{S}\in R^{504\times 504}.\;\;\;\text{( similarity matrix) .} \end{array}\;$$

Each experiment has a multi-index **i** = (*i*_1_, *i*_2_), where *i*_1_ ∈ {1, …, 36} and *i*_2_ ∈ {1, …, 14}. We compute the 504 × 504 cosine similarity matrix $\tilde{S}$ of the normalized rows of $\tilde{Z}$ (see electronic supplementary materials, appendix, II.B and II.C).

## 4. Structured clustering

Given a similarity tensor **S**, we seek the best partition of experiments subject to the interpretability constraints: clusters must be rectangular with respect to cell lines and ligands (see equation (4.1) and results section). This approach is similar to those in [7]; however, we do not require the rectangles to be connected. This is because we do not require a fixed order for the rows and columns of the data. This is an important strength of our method: an ordering of the data is artificial, and we seek clustering results that are not biased by order.

We present two implementations of our method. The first one does not require previous knowledge about the clustering assignment of the experiments, and provides an optimal clustering directly from the similarity data. However, owing to the high computational costs of performing integer programming, this variant of our method is only appropriate for small datasets. The computations can be sped up by employing heuristics for the integer optimization (e.g. [32]).

To tackle larger datasets, we present a second implementation that begins with a pre-existing partition of the experiments into clusters (not necessarily compliant with the constraints), which might originate from *any* clustering method (e.g. using the reshaped similarity tensor $\tilde{S}$). This implementation then reconstructs **S** and finds the nearest optimal clustering compliant with the constraints. Starting with an initial clustering has the advantage that we can employ the best methods for clustering a particular type of data, whose results we then refine to find clusters that are compatible with the interpretability condition. The initial clustering must be chosen carefully to fit the application, and should not be viewed as merely an initialization of the algorithm. Pairing our method with a pre-existing clustering also has the advantage that it significantly reduces computational cost (see electronic supplementary material, appendix, figure S7).

### 4.1. No prior clustering

When we do not have any prior clustering of the experiments, we work directly on the similarity tensor **S**. The entries of this tensor record the similarity of experiments **i** and **j**, where **i** = (*i*_1_, *i*_2_), **j** = (*j*_1_, *j*_2_), where the ranges of indices are *i*_1_, *j*_1_ ∈ {1, …, 36} and *i*_2_, *j*_2_ ∈ {1, …, 14}.

The clustering assignments are recorded by the tensor **X** defined in equation (2.2). The rectangular-shaped interpretability condition corresponds to three types of algebraic constraints on the entries of **X**,

4.1 $$\left.\begin{array}{rl} & x_{i_{1}i_{2}j_{1}j_{2}}=x_{i_{1}j_{2}j_{1}i_{2}},\\ & 0\leq x_{i_{1}i_{2}j_{1}j_{2}}-x_{i_{1}i_{2}j_{1}i_{2}}\leq 1\\ \mathrm{and}\;\;\; & 0\leq x_{i_{1}i_{2}j_{1}j_{2}}-x_{i_{1}i_{2}i_{1}j_{2}}\leq 1. \end{array}\right\}$$

We search over arrays **X** that satisfy these conditions. The experiments in the same cluster should have high similarity, so we maximize the similarity between experiments in the same cluster. This maximization is equivalent to solving the integer optimization problem

4.2 $$\begin{array}{rl} \max_{X} & \;\langle S,(1-X)\rangle +\lambda \langle 1,X\rangle ,\\ \mathrm{subject}\;\mathrm{to} & \;b_{l}\leq V\cdot \mathrm{vec}(X)\leq b_{u}, \end{array}$$

where the tensors **X** and **S** are as above, ⟨⋅,⋅⟩ denotes the entry-wise inner product and · represents matrix multiplication of the matrix **V** by the vector vec(**X**). The 504^2^ × 1 vector vec(**X**) is the vectorized form of **X**, and **1** is the tensor of 1s with the same size as **X**. The coefficient *λ* is a regularization term introduced to control the number of clusters. The matrix **V** encodes the constraints on **X** given in equations (2.3) and (4.1). This matrix has over 1 million rows, 504^2^ columns and is extremely sparse. The *k*th row of **V** represents the *k*th constraint on the values of vec(**X**): the entry is the coefficient (which can be 0, 1 or −1) with which each entry of vec(**X**) appears in the constraint. The *k*th entry of *b*_*l*_ and *b*_*u*_ (which can be 0, 1 or 2) gives the lower and upper bounds, respectively, of each linear inequality. We solve this optimization program using the branch and cut algorithm [31] via the IBM ILOG CPLEX Optimization Studio [33].

The resulting rectangular clusters are a sparse, low-rank representation of the data. The tensor **1** − **X**, of size (36 × 14) × (36 × 14), gives a binary measure of the distance between any two experiments. This tensor has sparse block structure: it consists of *m* cuboids of 1s along the diagonal, where *m* is the number of clusters, and has zeros everywhere else. As a consequence **X** has low multilinear rank [34], bounded above by (*m*, *m*, *m*, *m*), which is less than the maximum possible value of (36, 14, 36, 14).

### 4.2. Pre-existing clusters

When we have a pre-existing or initial non-rectangular clustering of the experiments, we find the nearest structured clusters using linear integer optimization. The input to this method is an initial partition of the 504 experiments into *m* clusters. We then modify the cluster assignments to reach the closest possible interpretable, structured clustering.

The initial clustering is encoded by a partition tensor, **W**, of size 36 × 14 × *m*

$$w_{ik}=\left\{\begin{array}{cc} 1, & i\;\text{is in cluster}\;k,\\ 0, & \text{otherwise},\; \end{array}\right.$$

where **i** = (*i*_1_, *i*_2_) indexes an experiment. The new clusters are encoded by a tensor **Y** of the same size (defined according to equation (2.4)). In order to have rectangular clusters, the entries of **Y** must satisfy the conditions

$$\begin{array}{rl} \sum_{r=1}^{m}y_{ijr}=1\; & \;\text{( unique cluster assignment)}\\ \mathrm{and}\;-1\leq y_{ikr}+y_{\;jlr}-y_{ilr}\leq 1 & \;\text{( interpretability condition)}. \end{array}\;$$

As before, we use the branch and cut algorithm to obtain the global optimum (given **W**) for the optimization problem

4.3 $$\max_{Y}\;\langle W,Y\rangle .$$

The inner product 〈**W**, **Y**〉 sums the number of clustering assignments unchanged by converting the initial unstructured clustering into a clustering that satisfies the interpretability constraints.

We obtain the tensor **Y**, of size 36 × 14 × *m* by solving the optimization problem in equation (4.3). As with **X**, the tensor **Y** also has sparse and low-rank structure. Its *m* two-dimensional slices, each a matrix of size 36 × 14, have rank 2 and block structure with a rectangular shape populated by 1s and all other values equal to 0.

## 5. Results

### 5.1. Biological interpretation of constraints

Each experiment in our data is indexed by (*c*_*i*_, *l*_*j*_), where *c*_*i*_ is the *i*th cell line and *l*_*j*_ is the *j*th ligand. A high similarity between experiments suggests the possibility of a common underlying biological mechanism. This is the basic notion that underpins the constraints in our clustering method, which force the clusters to pair a subset of the cell lines with a subset of the ligands in such a way that each cluster must be rectangular, although possibly disconnected (figure 2). The motivation behind this constraint is to enable the interpretation that the experiments in each cluster are generated by the same biological mechanism (e.g. if they share a feature such as a genetic mutation). The difference between our constrained approach and conventional clustering is that in the latter a high similarity is enough to cluster two experiments together. In our approach, similarity alone is not enough, we also require that the observations admit the same mechanistic interpretation. For example, suppose that two experiments (*c*_*h*_, *l*_*i*_) and (*c*_*k*_, *l*_*j*_) belong to the same cluster. If we swapped the ligands (i.e. we looked at the experiments in the diagonally opposite entries (*c*_*k*_, *l*_*i*_) and (*c*_*h*_, *l*_*j*_)), under the assumption that the cell lines share the same signalling mechanism, these experiments should also be in the same cluster because we expect them to respond in a similar way (see left columns of figure 2). If, however, (*c*_*h*_, *l*_*i*_) and (*c*_*k*_, *l*_*j*_) are clustered together but (*c*_*k*_, *l*_*i*_) and (*c*_*h*_, *l*_*j*_) are not in the same cluster (see right column of figure 2), it would be more difficult to assign mechanistic interpretations to the clusters.

### 5.2. Interpretable groups by mutation and receptor subtype

In a clinical setting, prognosis and treatment decisions for breast cancer are guided by tumour grade, stage and clinical subtype (see http://www.cancer.gov) which is based on the presence of cellular receptors:

— HER2^amp^ cells are characterized by amplification of the HER2 gene, leading to over-expression of the ErbB2 receptor tyrosine kinase;
— HR^+^ cells are characterized by the expression of the oestrogen receptor (ER) or progesterone receptor (PR);
— triple negative breast cancer (TNBC) cells are negative for HER2 amplification, and express ER and PR at low levels.

We compare the clusters from our method with the three standard clinical subtypes above. We also compare our clusters with the mutational status of the cell lines [35,36], and with their drug response [37,38], and with the findings from the previous clustering and analysis of this dataset, found in [26].

**Figure 3.**
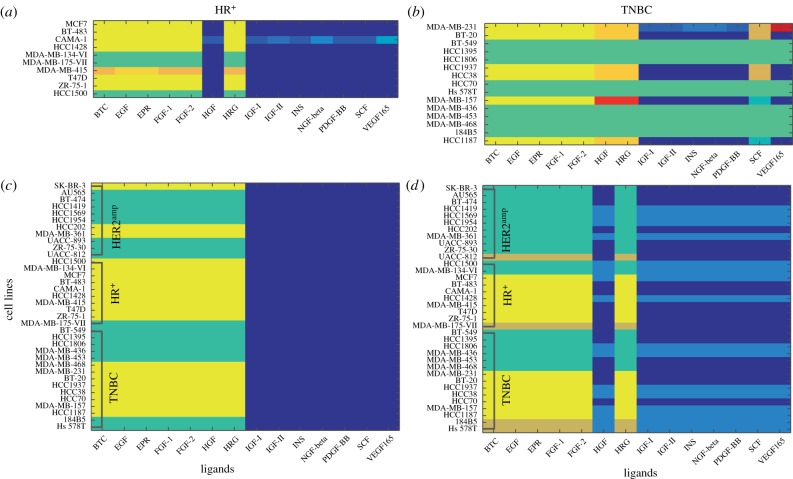
Tensor-based structured clustering. (*a*) TNBC clustering with no prior clustering information. (*b*) HR^+^ clustering with no prior information. (*c*) Clustering of all cell lines starting from an initial partition into three clusters. (*d*) Clustering from an initial partition into five clusters. Note that the colours on the grid represent clustering assignments, and are not reflective of the intensity of any single parameter. (Online version in colour.)

We first investigate a fine-grained classification within each of the three clinical subtypes. A summary statistic between 0 and 1 (based on the cosine similarity; see electronic supplementary material, appendix II.B) quantifies the within-class variation for each clinical subtype. A score of 0 indicates complete homogeneity, and 1 indicates complete heterogeneity. The HER2^amp^ cell lines show comparatively little variation, with an average difference score of 0.086. The TNBC and the HR^+^ cell lines have an average difference score of 0.224 and 0.334. We obtained clusters without prior knowledge of an initial clustering by solving the optimization problem (4.2). The results (shown in figure 3*a*,*b*) identify heterogeneity within each subtype as well as cell lines of particular interest.

Figure 3*a* shows the clustering of the HR^+^ cell lines. Cell line MDA-MB-415 stands out for its response to the so-called high-response ligands [26] (ligands to the left of HRG in figure 3*b*). Among all cell lines, MDA-MB-415 has the second highest susceptibility to the drugs ixabepilone, methylglyoxal and PD [37]. The CAMA-1 cell line is distinctive in its response to the low-response ligands (to the right of HRG), which might help explain why it is particularly susceptible to both (Z)-4-hydroxytamoxifen and TCS PIM-11 [37]. The TNBC cell lines are divided into 12 clusters (figure 3*b*), which mirror the heterogeneous behaviour of TNBC in the clinic [39]. All but one TNBC cell lines with a PTEN mutation appear in the green cluster. The only exception is the HCC1937 cell line, which has a PTEN mutation but appears in the yellow cluster. The cluster assignment of cell lines MDA-MB-231 and MDA-MB-157 is markedly different from that of the other cells across the ligands. These assignments might be explained by the mutational status of the cell lines; MDA-MB-231 is the only cell line with an NF2 mutation or a BRAF mutation, whereas MDA-MB-157 is the only cell line with an NF1 mutation. The bright orange cluster contains five cell lines (all but HCC1937) with the same two CDKH2A mutations.

The HER2^amp^ cell lines cluster together for all ligands except for the MDA-MB-361 cell line. This is the HER2^amp^ cell line most resistant to HER2-targeted therapy such as lapatinib [37]. In fact, its resistance to lapatinib exceeds that of some TNBC cell lines (HCC2185 and MDA-MB-453). The grouping of the rest indicates the consistency among all other HER2^amp^ cell lines (see electronic supplementary material, appendix II.B).

### 5.3. Clustering all cell lines

To cluster all cell lines, we solve the optimization problem (4.3), which requires an initial ‘seed’ clustering of the experiments. We obtained our initial clustering by first constructing a graph of experiments from the similarity matrix $\tilde{S}$ using the relaxed minimum spanning tree algorithm [40–42]. Then we used the Markov stability community detection method [43,44] to find robust partitions of the experiments into three, five and seven groups (see electronic supplementary material, appendix, figure S3).

From the initial partition into three clusters, we obtain three rectangular clusters (figure 3*c*). These groups respect the broad division of the cell lines seen in figure 3*a*,*b*, which is a sign of the consistency between the two implementations of our method. Of these, we find that two groups of ligands correspond to previously reported high active expression profiles (yellow and green) and one to muted profiles (blue) [26]. Within the more highly active group, the HR^+^ cell lines are predominantly in the yellow cluster, while the HER2^amp^ cells are in the green cluster. This separation of the HR^+^ and HER2^amp^ clinical subtypes is entirely data driven and supports the notion that our method is indeed able to find interpretable groups. The cell lines that are not clustered according to their subtype reflect previous findings that neither growth factor responses nor sensitivity to drugs that target signal transduction pathways is uniform within clinical subtypes [26,28,45]. The TNBC cell lines are divided between the yellow and green clusters, providing further evidence of the heterogeneity in TNBC cell lines [45–50].

When we start from the initial non-rectangular clustering into five groups, the resulting rectangular clusters split the ligands into a low response group (blues) and high response (green, yellow, brown). This split is nearly the same as we obtained before (figure 3*d*). Note that the difference in the ligand HGF may be due to the fact that it is not part of the ErbB nor the FGF families of ligands. The HER2^amp^ cell lines are now all assigned to the green cluster, and there are only three HR^+^ cell lines not assigned to the yellow cluster. A new brown cluster consists of cell lines: MDA-MB-175-VII (classified as a HR^+^), UACC-812 (HER2^amp^), 1845B5 (TNBC) and HS578T (TNBC). While none of them has the same cell classification or genetic mutation, all cell lines in the brown cluster show high susceptibility to the drug gefitinib [37]. Note that MDA-MB-175-VII is the only HR^+^ cell line that is not assigned to the yellow group in either three or five clusters; this might be due to the fact that this cell line carries a unique chromosomal translocation. The translocation leads to the fusion and amplification of neuregulin-1, which signals through ErbB2/ErbB3 heterodimers [51,52], and could be the underlying cause of the cell line’s unique sensitivity to ErbB-targeting drugs such as lapatinib or afatinib [28,45].

**Figure 4.**
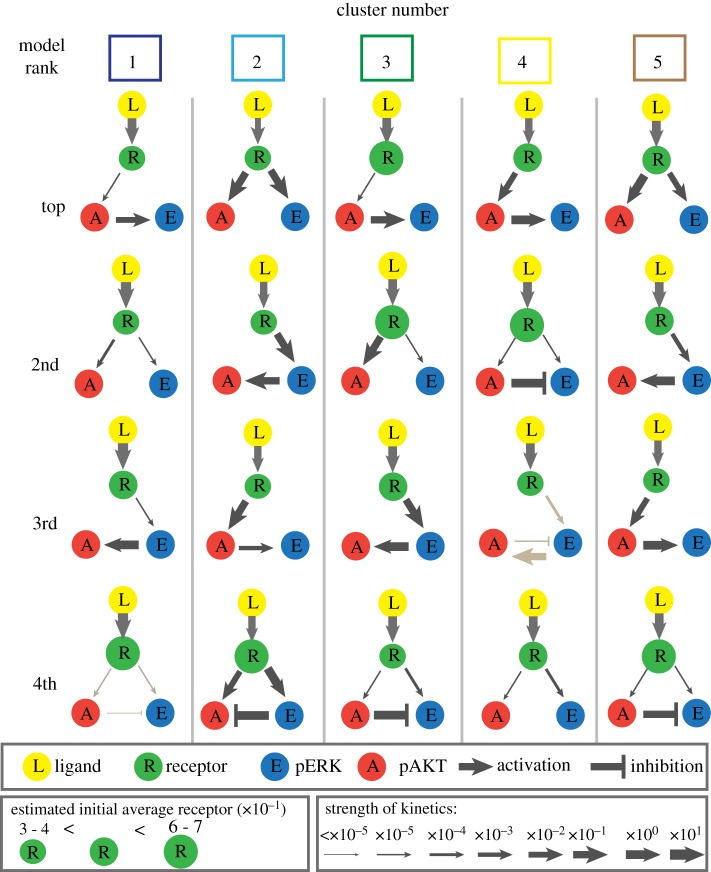
The top four models for each cluster according to the AICc ranking. The strength of interaction is indicated by the size of the arrow. The grey arrows indicate a blocking mechanism for inhibition. Black inhibition arrows indicate a removal mechanism for inhibition. (Online version in colour.)

We compare the results from our clustering method with the original analysis of this dataset [26]. In our analysis, we are able to obtain simultaneously meaningful subsets of *both indices* (the cell lines and the ligands), without biases given to either index or to the ordering of the data. By contrast, in the unstructured clusters shown in [26, fig. 3], the interpretation of the results required aggregating information to study how the effects vary with each cell line or with each ligand individually, but not simultaneously [26, fig. 4]. Our method allowed the clinical subtypes to be recovered from the data, based on temporal responses to a detected subset of the ligands. Exceptions to this classification provide biological hypotheses for possible subsequent investigation. By contrast, the clinical subtypes were not detectable from the clustering assignments of the temporal data made in [26, fig. 3].

The clustering that begins from an initial partition into seven groups shows high consistency with the five cluster case (see electronic supplementary material, appendix, figure S6). We therefore continue our analysis on the five rectangular clusters.

### 5.4. Systematic model identification

We now analyse the response of the five structured groups found in the previous section (figure 3*d*) to obtain a mechanistic insight about the cell line/ligand combinations in each cluster. We consider 729 possible ODE network models, and then perform systematic model analysis of the 44 that are structurally identifiable with the given data. We test structural identifiability, a prerequisite for performing parameter estimation and model selection, using Daisy [53]. Then, we parametrize, rank and choose the models that best represent each cluster’s response. As a result, we have a list of candidate signalling mechanisms for each cluster which provides more information than the statistical predictions of the sensitivity of MAPK drug targets (e.g. ErbB drug class) [45].

Models of the MAPK and AKT pathways have been studied under a variety of biological and modelling assumptions [54–56], including pathway crosstalk [20,21,57,58]. Here we consider simple models to ensure the parameters are at least locally identifiable so there are a finite number of parameter values to fit the data. We construct nonlinear ordinary differential equation models to describe the dynamics of the AKT and ERK signalling pathways. See the electronic supplementary material, appendix, for a synopsis of MAPK models and details of their construction. Briefly, these models include three molecular species: receptor (R), pERK (E) and pAKT (A). Since the data contain the response of pERK and pAKT, we assume that the receptor must phosphorylate ERK and/or AKT. We consider positive, negative or no interaction between pERK and pAKT under different types of kinetic regimes (mass action or Michaelis–Menten) and different types of inhibition (blocking/sequestration or removal/degradation). The combination of these features results in the 44 structurally identifiable models that we study in further detail. Each model corresponds to a different mechanistic hypothesis of the dynamics in the pathways (see electronic supplementary material, appendix III.C). To find the models that best describe the response of each of the five clusters, we estimate parameters using the squeeze-and-breathe algorithm [59], and rank them using the Akaike information criterion score (AICc) (see electronic supplementary material, appendix IV.D). The best models for each cluster are shown in figure 4.

The AICc, used for model selection, penalizes more complex models; therefore, it is not surprising that the top models are the simplest ones. The best models for each cluster have different feedback strengths (parameter values) and network topologies (figure 4); this supports the hypothesis that mutations may play a role in the dynamics. Although the values of the parameters vary, the model with arrows from the receptor (R) to pAKT and pERK appears in all clusters, which is in line with how cells are understood to operate. We remark that cluster 4, which corresponds to HR^+^ cells (yellow in figure 2*d*), includes inhibition crosstalk as the second best model, whereas in all other clusters this mechanism appears in fourth place. This finding suggests the possibility that the cell lines in cluster 4 share a feature which is relevant to the ligands that also appear in this cluster. This type of insight is made possible because of the constraint we have imposed on the clusters.

## 6. Discussion

We have introduced a novel framework to cluster multi-indexed data based on tensors that allows structural constraints to be incorporated using algebraic relationships. This method can be used to extract clusters directly from the data, and, if an initial clustering which may not satisfy the constraints is provided, it can find the closest optimal partition that satisfies the constraints. A key advantage of this framework is that it allows more control over the composition of clusters than in many unsupervised methods, and allows the clustering to be tailored to the requirements of the problem. The main limitation of this method is that it requires *complete* data (i.e. a measurement for every cell line/time/dose/ligand/molecule combination), which can be difficult to obtain. The metric used to compare data points could be adapted to deal with a small number of missing entries, but the method is unlikely to perform well for sparse data.

We applied this method on a dataset charting the response of genetically diverse breast cancer cell lines to ligands. We identified both similarities (e.g. HER2^amp^) and heterogeneities (e.g. TNBC) within clinical subtypes. The heterogeneity of our clustering analysis (figure 3*b*) seems to be related to both the mutational status of the cells as well as their response to inhibitors. This result means that similar analyses in patient tissues might be able to identify patients that respond differently to therapeutic methods commonly used within a clinical subtype. By analysing clusters from all subtypes, we also showed that we cannot attribute the dynamics of each data cluster with only one signalling mechanism, which helps explain network model differences across cell type.

The applicability of our method goes beyond the biological problem presented here. It can be used in any context in which the constraints on the clusters can be expressed in algebraic form (as equalities and inequalities), such as when there are size restrictions on the clusters, or to impose/prohibit particular combinations of data beyond *must-link* and *cannot-link* constraints. For example, this method could be used to construct optimal portfolios that comply with rules about their composition [60], to help the formation of teams that maximize members’ preferences and are compliant with skill requirements [61] and to find communities in networks with quotas, among others. The presented pipeline (a sophisticated and interpretable data analysis method that feeds into a nonlinear modelling framework) will be ever more necessary as increasingly more large-scale, comprehensive datasets become available.

## Supplementary Material

Supplementary Information for: Tensor clustering with algebraic constraints gives interpretable groups of crosstalk mechanisms in breast cancer

## Data accessibility

The dataset used in this analysis can be found in [26]; we also give a link to the data in the electronic supplementary material. The Matlab code for the constrained clustering method can be found in the electronic supplementary material, Section B.5 ‘Code for constrained clustering’. A link to the code for the mechanistic analysis can be found in the electronic supplementary material, Section D.3 ‘Parameter estimation’.
