# Supplementary material for: Tensor clustering with algebraic constraints gives interpretable groups of crosstalk mechanisms in breast cancer
Source: J R Soc Interface. 2019 Feb 6;16(151):20180661. doi: 10.1098/rsif.2018.0661 (PMC6408352; doi:10.1098/rsif.2018.0661)
Supplement: Supplementary Information for: Tensor clustering with algebraic constraints gives interpretable groups of crosstalk mechanisms in breast cancer [file rsif20180661supp1.pdf]

Supplementary Information for: Tensor clustering with algebraic constraints gives interpretable groups of crosstalk mechanisms in breast cancer

Anna Seigal,[1,*] Mariano Beguerisse-Díaz,[2,†] Birgit Schoeberl,[3] Mario Niepel,[4] and Heather A. Harrington[2,‡]

[1] *Department of Mathematics, University of California, Berkeley, CA 94702, USA*
[2] *Mathematical Institute, University of Oxford, Oxford OX2 6GG, UK*
[3] *Novartis Institutes for BioMedical Research, Cambridge, MA 02139, USA*
[4] *Ribon Therapeutics, Lexington, MA 02421, USA*

(Dated: January 9, 2019)

Appendix A: Data

We analyse a data set first presented in Ref. [1], which is available for download at the webpage `http://lincs.hms.harvard.edu/niepel-bmcbiol-2014/`. The data consists of time course measurements (at 0, 10, 30, and 90 minutes) of the fold change in the phosphorylation levels of the mitogen activated protein kinase (MAPK) pERK and the phosphoinositide 3-kinase (PI3K) pAKT in 36 breast cancer cell lines (Table I), each exposed to two doses (low: 1 ng/ml and high: 100 ng/ml) of 14 different ligands (Table II). Figure S1 shows an example of the temporal response of pAKT and pERK in the cell line MCF7 to doses of betacellulin.

The data set is complete in the sense that it contains measurements of pAKT and pERK in every cell line, exposed to 2 doses of each of the 14 ligands at 4 time points. As a result, we are able to represent the data as a $\times 36 \times 14 \times 2 \times 3 \times 2$ tensor $\mathbf{Z}$, with entries z_{ijptd}. The index $p \in \{\text{pAKT, pERK}\}$ denotes which protein was measured, i corresponds to the cell lines in Table I, j corresponds to the ligands in Table II, $d \in \{1, 100\}$ denotes the doses, and $t \in \{10, 30, 90\}$ is time after the stimulus.

Appendix B: Clustering

We first describe how we normalize the experimental data for clustering. Then we provide details for how we clustered directly from the data, and then how we obtained and incorporated an initial clustering.

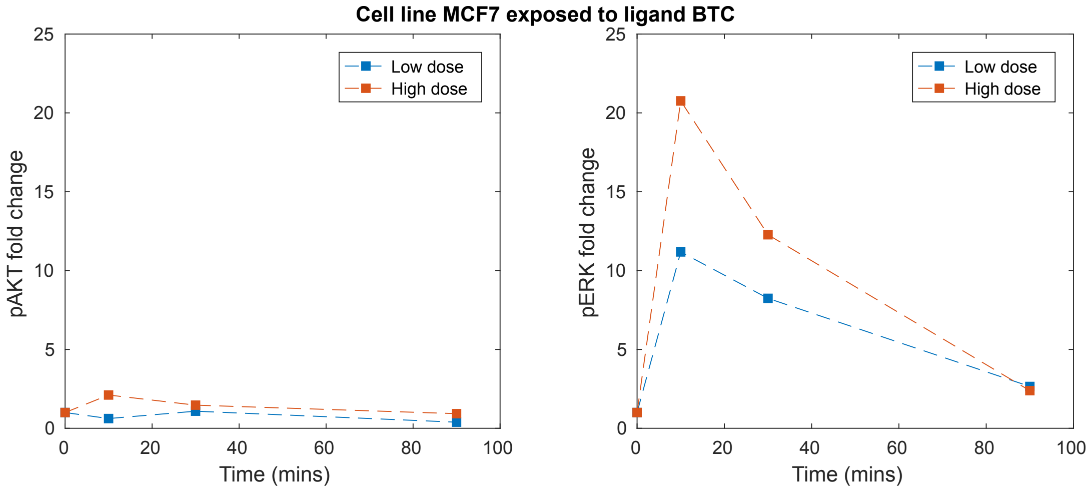

FIG. S1. Time course measurements of cell line MCF7 exposed to two doses of betacellulin (BTC).

* seigal@berkeley.edu, joint first author
† beguerisse@maths.ox.ac.uk, joint first author
‡ harrington@maths.ox.ac.uk

Cell line	Subtype
MCF7	HR+
SK-BR-3	HER2amp
MDA-MB-231	TNBC
AU-565	HER2amp
BT-20	TNBC
BT-474	HER2amp
BT-483	HR+
BT-549	TNBC
CAMA-1	HR+
HCC-1395	TNBC
HCC-1419	HER2amp
HCC-1428	HR+
HCC-1569	HER2amp
HCC-1806	TNBC
HCC-1937	TNBC
HCC-1954	HER2amp
HCC-202	HER2amp
HCC-38	TNBC
HCC-70	TNBC
Hs 578T	TNBC
MDA-MB-134VI	HR+
MDA-MB-157	TNBC
MDA-MB-175VII	HR+
MDA-MB-361	HER2amp
MDA-MB-415	HR+
MDA-MB-436	TNBC
MDA-MB-453	TNBC
MDA-MB-468	TNBC
T47D	HR+
UACC-812	HER2amp
UACC-893	HER2amp
ZR-75-1	HR+
ZR-75-30	HER2amp
184-B5	TNBC
HCC-1187	TNBC
HCC-1500	HR+

TABLE I. Breast cancer cell lines used in the data set [1].

Ligand name	Abbreviation
Betacellulin	BTC
Epidermal Growth Factor	EGF
Epiregulin	EPR
Fibroblast Growth Factor (acidic)	FGF-1
Fibroblast Growth Factor (basic)	FGF-2
Hepatocyte Growth Factor	HGF
Heregulin $\beta 1$	HRG
Insulin-like Growth Factor 1	IGF-1
Insulin-like Growth Factor 2	IGF-2
Insulin	INS
Nerve Growth Factor	NGF-beta
Platelet Derived Growth Factor BB	PDGF-BB
Stem Cell Factor	SCF
Vascular endothelial growth factor A	VEGF165

TABLE II. Ligands used in the data set [1].

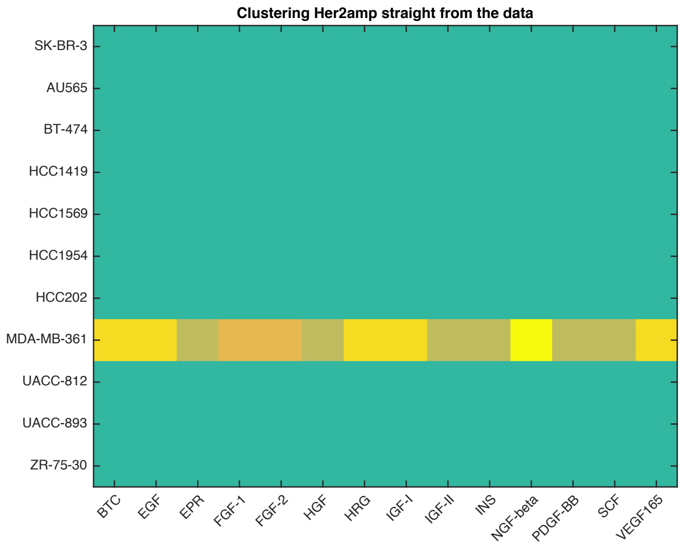

FIG. S2. Clusters from the data of HER2^{amp} cell line.

1. Normalization

We normalize the data as follows. We scale all the pAKT and pERK foldchange responses such that their average takes each the value 1. This normalization balances the effects of AKT and ERK, so that the behavioural features and not the scale are the dominating features, and to ensure we treat them with equal significance in our study. The mean value (pre-normalization) across the AKT responses is 1.7754 and that across the ERK responses is 11.4190.

2. No prior clustering

As a summary statistic of the three clinical subtypes, we compute the average distance score within each subtype. The score for the 11 HER2^{amp} cell lines is obtained as follows. There are $154 = 11 \times 14$ experiments in the dataset that involve HER2^{amp} cell lines, each consisting of 12 measurements. For each pair of experiments, we find the dissimilarity between the 12 measurements, using cosine dissimilarity

$$1 - \frac{\langle v_1, v_2 \rangle}{\|v_1\|_2 \|v_2\|_2},$$

where $\langle \cdot, \cdot \rangle$ is the usual inner product in a real vector space and $\| \cdot \|_2$ is the Euclidean norm. The average dissimilarity is obtained by averaging these pairwise distances across the $\binom{154}{2} = 11781$ pairs. Similarly for the HR^+ cell lines and for the TNBC cell lines. The averages obtained are 0.086 for HER2^{amp}, 0.334 for HR^+ and 0.224 for TNBC. The partitioning of the HER2^{amp} cell lines is given in Fig. S2.

3. Pre-existing clusters

a. Computing pre-existing clusters

We find an initial clustering of the experiments. For this initial clustering, we label each experiment by a single index. The data for the ith experiment is:

$$\widetilde{\mathbf{Z}}(i, :) = \left[\mathbf{AKT}_i^1 \middle| \, \mathbf{ERK}_i^1 \middle| \, \mathbf{AKT}_i^{100} \middle| \, \mathbf{ERK}_i^{100} \right], \tag{B1}$$

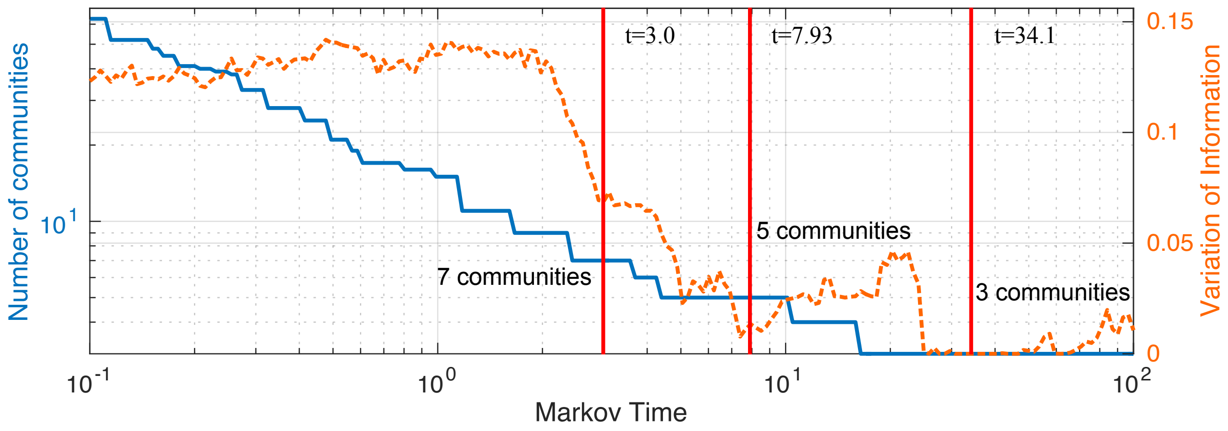

FIG. S3. Number of communities and variation of information for the network obtained from RMST similarity graph.

where $\mathbf{AKT}_i^1$ is the normalised time series of fold-change response of pAKT under dose 1ng/ml, and so on. We compute the 504×504 similarity matrix $\widetilde{\mathbf{S}}$, in which s_{ij} indicates the cosine similarity of experiments i and j:

$$s_{ij} = \frac{\langle \tilde{\mathbf{z}}_i, \tilde{\mathbf{z}}_j \rangle}{||\tilde{\mathbf{z}}_i||_2 \, ||\tilde{\mathbf{z}}_j||_2} = \cos(\tilde{\mathbf{z}}_i, \tilde{\mathbf{z}}_j). \tag{B2}$$

where $\tilde{\mathbf{z}}_i = \widetilde{\mathbf{Z}}(i,:)$ and $\tilde{\mathbf{z}}_j = \widetilde{\mathbf{Z}}(j,:)$. The entries of $\tilde{\mathbf{z}}_i$ and $\tilde{\mathbf{z}}_j$ are nonnegative, which means that $s_{ij} \in [0,1]$. If $s_{ij} = 1$, experiments i and j have an *identical* response to the treatments in both AKT and ERK (up to a scaling constant). When $s_{ij} = 0$, the data for the experiments are orthogonal. The task of clustering the experiments faces two challenges: the number of clusters is not known a priori, and the matrix $\widetilde{\mathbf{S}}$ is full matrix and is noisy due to experimental error. To tackle these challenges we use a combination of tools from manifold learning and network science. We create a network (graph) in which each of the 504 experiments is represented by a node, and where connections exist between similar experiments. We first define the dissimilarity matrix $\mathbf{D}$ ($d_{ij} = 1 - s_{ij}$). We then use the *Relaxed Minimum Spanning Tree* (RMST) algorithm [2–4], which extracts a network representation from high-dimensional point clouds (in this case the $\tilde{\mathbf{z}}_i$) that are embedded in a lower dimensional manifold.

Specifically, the algorithm creates an undirected, unweighted network with an edge between i and j if they are neighbors in a minimum spanning tree (MST) from $\mathbf{D}$. The algorithm adds extra edges to the network if they are consistent with the continuity of the data, i.e. if the distances between the points in $\mathbf{D}$ is comparable to their separation in the MST and is consistent with the continuity of the data, according to the equation

$$d_{ij} < \mathrm{mlink_{ij}} + \frac{1}{2}(\mathrm{k_i} + \mathrm{k_j}).$$

Here $\mathrm{mlink_{ij}}$ is the maximal edge weight in the MST path connecting i to j, and k_i is the the distance to the nearest neighbour of i (i.e., the minimum value on the ith row of $\mathbf{D}$, excluding d_{ii}). Basically, what the RMST algorithm does is allows edges to be added to the MST (it 'relaxes' the MST), so that we obtain an network description of high dimensional data that is embedded on a lower dimensional manifold.

Once we have obtained the network from the similarity matrix, we extract communities using the Markov Stability (MS) community detection algorithm [5, 6]. This method employs continuous time random walks of varying duration (Markov time) to extract communities of the network at different levels of resolution. Shorter Markov times produce small communities, whereas longer Markov times lead to coarse partitions of the network. Obtaining the optimal partition of a network into communities is an NP complete problem, so MS uses heuristics to find communities. Because there is no guarantee of finding a global optimum, MS repeats the heuristic search 100 times for each Markov time. The variability in each set of 100 solutions is measured with the Variation of Information (VI) [7]. A low value of the VI for a Markov time indicates that the solutions obtained are similar to each other, we take this similarity as a sign that there is a robust partition of the network for this Markov time. In Fig. S3 we show that the network A has a robust partition into 3, 5 and 7 communities.

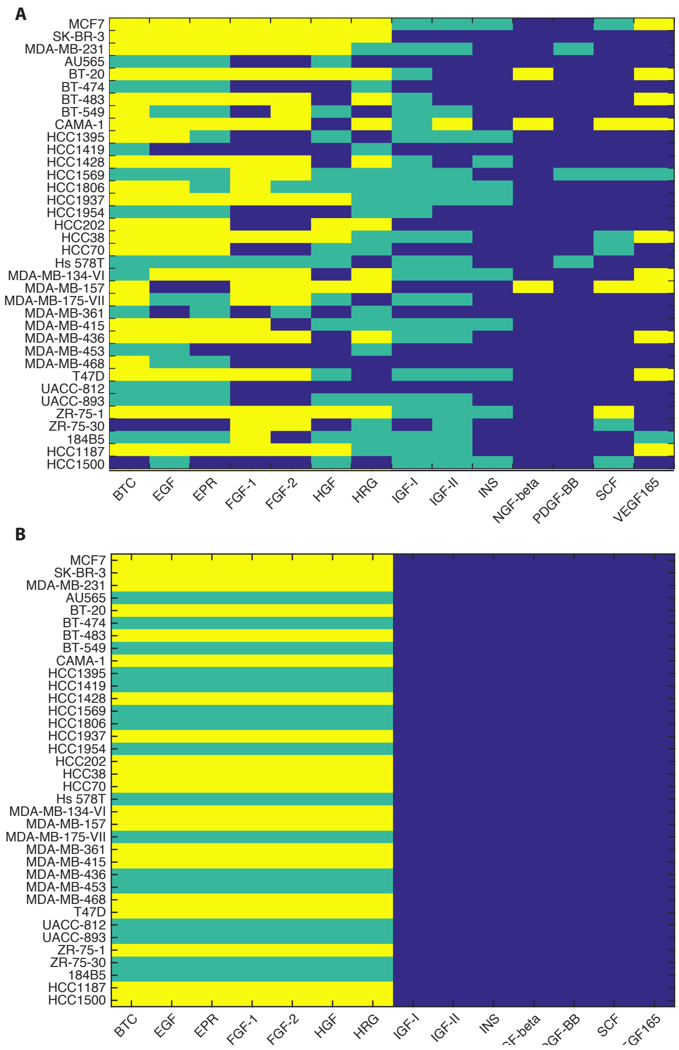

FIG. S4. A. The clustering assignments are represented by yellow, green and blue squares. B. The tensor clustering are a close approximation to the clusters obtained with MS.

b. Structured clusters from pre-existing clusters

We present the clustering assignments after using MS to obtain our initial partition into clusters. The pre-existing and structured cluster results for three clusters (Fig. S4), five clusters (Figs. 3D, S4) and seven clusters (Fig. S6).

A finer clustering into seven groups (see Fig. S6) divides the ligands into three groups: high response (yellow, green, brown) {BTC, EGF, EPR, FGF-1, FGF-2, HGF, HRG} and lower response (blues) {IGF1, IGF2, INS} and { NGF-β, PDGF-BB, SCF, VEGF175}. The assignment of cell types is remarkably similar to the five cluster results. The exception cell lines are: HCC1419 (green to brown), and ZR-75-30 (green to the new seventh cluster). Given this consistency, we restrict to mechanistic interpretation of the five cluster case.

4. Comparison of implementations

When we employ our method assuming no initial clusterings, the integer optimization is learning the values, 0 or 1, for an array of size $c \times c \times l \times l$ (where c = #cell lines and l = # ligands). When we use an initial clustering, CPLEX uses the same branch and cut algorithm on an array of size $c \times l \times k$, where c = #cell lines, l = #ligands and k = #clusters, so the array is much smaller for the pre-existing clustering implementation. We show a comparison of the performance of the two implementations in Fig. S7

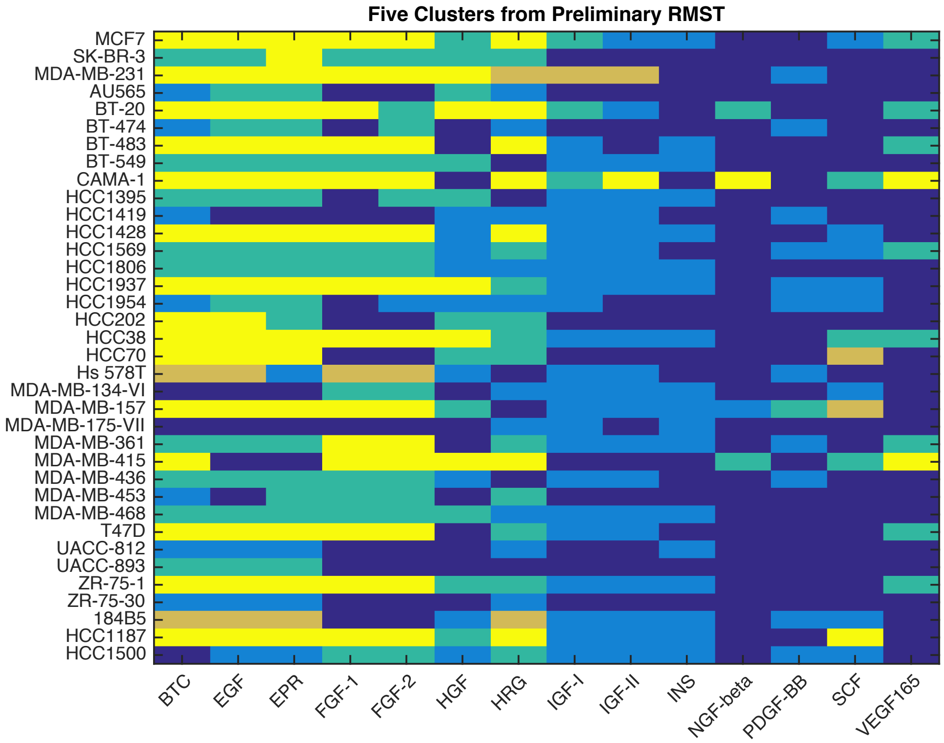

FIG. S5. Five clusters from Markov Stability

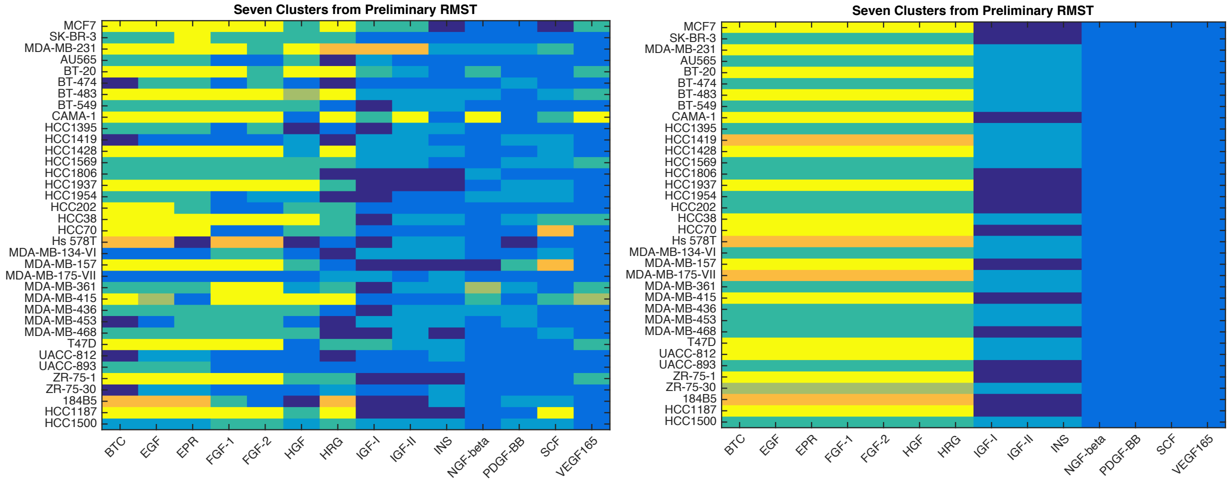

FIG. S6. Seven clusters from Markov Stability.

5. Code for constrained clustering

The following pieces of Matlab code generate the constraints on the tensors that encode the clustering assignment, under the rectangle clusterings condition. Assume we have a dataset of size $n \times m \times \cdots \times o$ and we wish to cluster into rectangular-shaped clusters using the first two indices. First, reshape the data into a three-dimensional array, `Tensr`, by vectorizing the 3rd, 4th, …, indices into a multi-index of size p. Then, the following code makes the similarity tensor with respect to cosine dissimilarity.

```
[n m p] = size(Tensr);
C = zeros(n,n,m,m);
for i = 1:n; for j = 1:n; for k = 1:m; for l = 1:m;
```

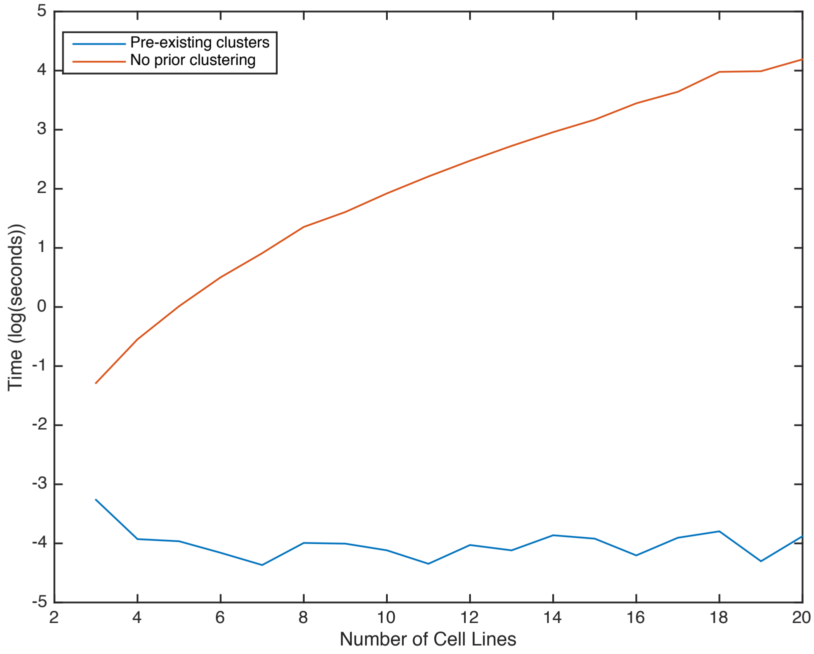

FIG. S7. Computational complexity of two implementations with eight ligands and a varying number of cell lines.

```
                v1 = zeros(p,1); v2 = zeros(p,1);
                for ii = 1:p;
                    v1(ii) = Tensr(i,k,ii); v2(ii) = Tensr(j,l,ii);
                end
                C(i,j,k,l) = 1 - dot(v1,v2)/(norm(v1,2)*norm(v2,2));
end end end end
```

Next we encode the constraints on the tensor which encodes the partition of the data set. It is constructed as a sparse array, i.e. by specifying row and column indices `Vrow` and `Vcol`, and values `Vval` of all non-zero entries. The lower and upper bounds for each linear constraints are organized into the vectors `lb` and `ub` respectively.

```
t = 1;
for i = 1:n; for j = 1:n; for k = 1:m; for l = 1:m;
                if (i ~= j) || (k ~= l);
                    W = vectorize(n,i,j,k,l);
                    Vrow(w) = t; Vcol(w) = W;Vval(w) = 1;
                    w = w+1;
                    W = vectorize(n,j,i,l,k);
                    Vrow(w) = t; Vcol(w) = W; Vval(w) = -1;
                    w = w+1;
                    lb(t) = 0; ub(t) = 0;
                    t = t+1;
                end
                if (k ~= l);
                    W = vectorize(n,i,j,k,l);
                    Vrow(w) = t; Vcol(w) = W; Vval(w) = 1;
                    w = w+1;
                    W = vectorize(n,i,j,l,k);
                    Vrow(w) = t; Vcol(w) = W; Vval(w) = -1;
                    w = w+1;
                    lb(t) = 0; ub(t) = 0;
                    t = t+1;
                % opposite diagonals must be same
                end
```

```
                if (i ~= j);
                    W = vectorize(n,i,j,k,l);
                    Vrow(w) = t; Vcol(w) = W; Vval(w) = 1;
                    w = w+1;
                    W = vectorize(n,j,i,k,l);
                    Vrow(w) = t; Vcol(w) = W; Vval(w) = -1;
                    w = w+1;
                    lb(t) = 0; ub(t) = 0;
                    t = t+1;
                end
                if (k ~= l);
                    W = vectorize(n,i,j,k,l);
                    Vrow(w) = t; Vcol(w) = W; Vval(w) = 1;
                    w = w+1;
                    W = vectorize(n,j,i,k,k);
                    Vrow(w) = t; Vcol(w) = W; Vval(w) = -1;
                    w = w+1;
                    lb(t) = 0; ub(t) = 1;
                    t = t+1;
                % vertical conditions
                end
                if (i ~= j);
                    W = vectorize(n,i,j,k,l);
                    Vrow(w) = t; Vcol(w) = W; Vval(w) = 1;
                    w = w+1;
                    W = vectorize(n,i,i,k,l);
                    Vrow(w) = t; Vcol(w) = W; Vval(w) = -1;
                    w = w+1;
                    lb(t) = 0; ub(t) = 1;
                    t = t+1;
                % horizontal conditions
                end
end end end end

for i = 1:n; for k = 1:m;
        W = vectorize(n,i,i,k,k);
        Vrow(w) = t; Vcol(w) = W; Vval(w) = 1;
        w = w+1;
        lb(t) = 0; ub(t) = 0;
        t = t+1;
end end

for i1 = 1:n; for i2 = 1:n; for i3 = 1:n;
for k1 = 1:m; for k2 = 1:m; for k3 = 1:m;
if (((i1 ~= i2) || (k1 ~= k2)) && ((i2 ~= i3) || (k2 ~= k3)) && ((i1 ~= i3) || (k1 ~= k3)));
             W = vectorize(n,i1,i3,k1,k3);
             Vrow(w) = t; Vcol(w) = W; Vval(w) = -1;
             w = w+1;
             W = vectorize(n,i1,i2,k1,k2);
             Vrow(w) = t; Vcol(w) = W; Vval(w) = 1;
             w = w+1;
             W = vectorize(n,i2,i3,k2,k3);
             Vrow(w) = t; Vcol(w) = W; Vval(w) = 1;
             w = w+1;
             lb(t) = 0; ub(t) = 2;
             t = t+1;
end end end end end end end
maxt = t-1;
```

```
V = sparse(Vrow,Vcol,Vval,maxt,n*n*m*m);
```

Now complete the optimization using CPLEX, feeding the above constraints into the model alongside a penalty parameter that controls the number of clusters. The function `vectorize`, used above, combines the multi-index into a single index:

```
function t = vectorize(n,i,j,k,l);
m = 14; % for example
i1 = n*m*m*(i-1); j1 = m*m*(j-1); k1 = m*(k-1); l1 = l;
t = i1+j1+k1+l1;
```

For clustering based on pre-existing clusters, we generate the constraints imposed using the following Matlab code. Assume we are clustering based on 2 indices of sizes n and m, and that the preliminary clustering returned `nclu` clusters. As before, the lower and upper bounds on the conditions are organized into the vectors `lb` and `ub`.

```
w = 1; t = 1;

for i = 1:n; for j = 1:m;
for k = 1:nclu;
            W = nclu*m*(i-1) + nclu*(j-1) + k;
            % turn i,j,k coordinates into a vector
            Vrow(w) = t; Vcol(w) = W; Vval(w) = 1;
            w = w+1;
        end
        lb(t) = 1; ub(t) = 1;
        t = t+1;
end end
% the vector at position (i,j) has entries which sum to 1

for i = 1:n; for j = 1:n; for k = 1:m; for l = 1:m;
                if (i ~= j) && (k ~= l) ;
                for r = 1:nclu;
                    W = nclu*m*(i-1) + nclu*(k-1) + r;
                    Vrow(w) = t; Vcol(w) = W; Vval(w) = 1;
                    w = w+1;
                    W = nclu*m*(j-1) + nclu*(l-1) + r;
                    Vrow(w) = t; Vcol(w) = W; Vval(w) = 1;
                    w = w+1;
                    W = nclu*m*(i-1) + nclu*(l-1) + r;
                    Vrow(w) = t; Vcol(w) = W; Vval(w) = -1;
                    w = w+1;
                    lb(t) = -1; ub(t) = 1;
                    t = t+1;
                end end
end end end end
maxt = t-1;
% make sparse array of the constraints:
Vnew = sparse(Vrow,Vcol,Vval,maxt,n*m*nclu);
```

Appendix C: Models

1. Models of the MAPK pathway

The MAP Kinase pathway has been widely studied. Models have focused on various features of the cascade, such as its three-tier phosphorylation feedback structure [8, 9]. The activation profile of these kinases is directly related to cellular decisions and fates [10–13]. The AKT pathway has been modeled by EGF-dependent activation, including phosphorylation of AKT (pAKT) and its downstream intracellular proteins [14]. Few models of crosstalk between ERK and AKT exist. One model was created for studying PC12 cells, and they found that AKT acts as a low-pass

Number of arrows	Total number of networks	Number of networks we consider
All	$3^4 = 81$	15
0	$\binom{4}{0} \cdot 2^0 = 1$	None
1	$\binom{4}{1} \cdot 2^1 = 8$	None
2	$\binom{4}{2} \cdot 2^2 = 24$	3
3	$\binom{4}{3} \cdot 2^3 = 32$	8
4	$\binom{4}{4} \cdot 2^4 = 16$	4

TABLE III. In total, we consider 15 network topologies: those that are biologically plausible given the data. There are different possible kinetics for each model: mass-action or Michaelis-Menten. This gives $15 \times 2 = 30$ possible models. Furthermore, seven of these include inhibition (⊣) which we model via either: blocking or removal (described in the text). Accounting for all our kinetic models gives a total of 44 models.

filter which decouples the EGF signal [15]. Another model was created to study HEK293 cells in the presence of a MEK inhibitor; they found crosstalk is reinforced between Ras and PI3K [16]. Another model found that JNK is regulated by AKT and MAPK feedbacks in these pathway [17]. Chen and co-authors constructed and analyzed an ErbB model focusing on the receptor dynamics and early activation response of the MAPK and AKT pathways in response to ligands in A431 or H1666 cells [13]; however their model was unidentifiable, meaning there were an infinite number of parameter values to fit the data [13]. Weakly activated models of MAPK activation cascades with optimal amplification under a variety of stimuli were analyzed in [18].

2. All wiring diagrams

We consider all possible wiring diagrams to describe the interactions between the receptor, the Erk pathway and the Akt pathway. These can be written as a wiring-diagram where we assume an arrow exists between the ligand L and receptor R:

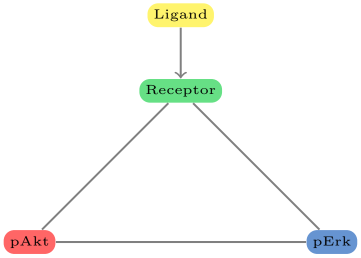

We first consider all possible network topologies with interactions between the variables R, E and A. There are three possibilities for the directed interaction from one variable to another: positive (→), negative (⊣), or no significant interaction (no arrow). There are six potential directed interactions in the network which give a total of $3^6 = 729$ networks.

Many of these networks can be ruled out. The data shows some response in pERK and pAKT for each stimuli, therefore we require that both pERK and pAKT have at least one arrow coming into each of these (to ensure a response). Given this restriction from the data, we can eliminate many networks, for example a network where the receptor inhibits pAKT and pERK is biologically infeasible. We also do not have dynamic data of the phosphorylated receptor, therefore we cannot distinguish a network topology that has an arrow feeding back from the phopho-form to the receptor; thus we fix the interaction from pERK to R and pAKT to R to none (no arrow). All of these restrictions produce Table III.

Based on these wiring diagrams, we now consider different possible kinetics for each arrow, summarized in the Table, and described in the next subsection.

3. Construction of mechanistic models

We construct systems of ordinary differential equation models to describe interaction dynamics between the receptor (R), pAKT (A) and pERK (E). The equation describing the evolution of the phosphorylated receptor is $dR/dt = \alpha L(R_{\text{tot}} - R) - \delta R$. The total amount of receptor, R_{tot}, is estimated from the receptor abundance data. The

unphosphorylated receptor is given by $(R_{\text{tot}} - R)$. The parameter α determines the rate at which it is phosphorylated by the ligand dose ($L = 1$ or 100 ng/ml). The time evolution of pERK and pAKT can be activated by R. There are two other equations, dE/dt and dA/dt, which describe the change in the phosphorylation (in fold change) of A and E with respect to time. Crosstalk between the ERK and AKT pathways is encoded by interactions between pERK and pAKT, which can either activate or inhibit the other pathway. Activation terms are modeled using either mass action or Michaelis-Menten kinetics. We consider two types of inhibition: blocking through a saturation term or through a removal term using mass action kinetics.

Without data from receptor dynamics, we write the change in phosphorylated receptor (in arbitrary units) as a function of time as:

$$R' = \alpha L(R_{tot} - R) - \delta R,$$

where $\alpha L(R_{tot} - R)$ describes the fraction of non-phosphorylated receptor that becomes phosphorylated at some rate proportional to the ligand L, and δ is the rate at which R is de-phosphorylated.

The other two equations, E' and A' describe the change in the phosphorylation (in fold change) of A and E with respect to time. These equations change based on the assumed interactions, each different set of equations describes a different mechanistic model.

We assume that activation is either via mass-action kinetics or Michaelis-Menten kinetics, and that inhibition is either via removal or blocking. For example, if we consider the model $R \to E \to A, \quad R \dashv A$, we can write this as:

$$R' = \alpha L(R_{tot} - R) - \delta R, \tag{C1}$$

$$E' = \frac{k_1 R}{K_{m1} + R} - \delta E, \tag{C2}$$

$$A' = \frac{k_2 E}{K_{m2} + E} - k_3 A R - \delta A, \tag{C3}$$

where the blue denotes the Michaelis-Menten term (ignoring the blue is mass-action), and the red term $k_3 AE$ describes inhibition of A as a removal interaction. However, when A is inhibiting by blocking, now the red term is written in a the following form:

$$R' = \alpha L(R_{tot} - R) - \delta R, \tag{C4}$$

$$E' = \frac{k_1 R}{K_{m1} + R} - \delta E, \tag{C5}$$

$$A' = \left(\frac{k_2 E}{K_{m2} + E}\right)\left(\frac{k_3}{k_3 + R}\right) - \delta A. \tag{C6}$$

We summarize the 44 models analyzed in more detail in Fig. S8.

Appendix D: Model identification

1. Estimating the total abundance of receptor, R_{tot}

Our mechanistic analysis requires us to estimate the total abundance of receptor (both phosphorylated and unphosphorylated) prior to the addition of any ligand. We do this based on the Receptor Abundance Data from [19, Figure 1]. Each ligand has one or more receptors associated to it, as shown in Table IV.

Each experiment involves a cell line and a ligand. For each cell line/ligand pair, we estimate the receptor abundance by averaging the receptor abundances for that cell line, for each of the receptors associated to the ligand. We do this averaging over all receptor, cell line pairs for which we have data.

We then estimate the receptor abundance for a cluster by averaging the values obtained above for each cell line, ligand pair that is present in the cluster.

2. Identifiability analysis

Before estimating model parameters from the data, we determine whether a model is identifiable. Models that are globally identifiable have parameters that are uniquely identifiable under ideal data conditions. Models that

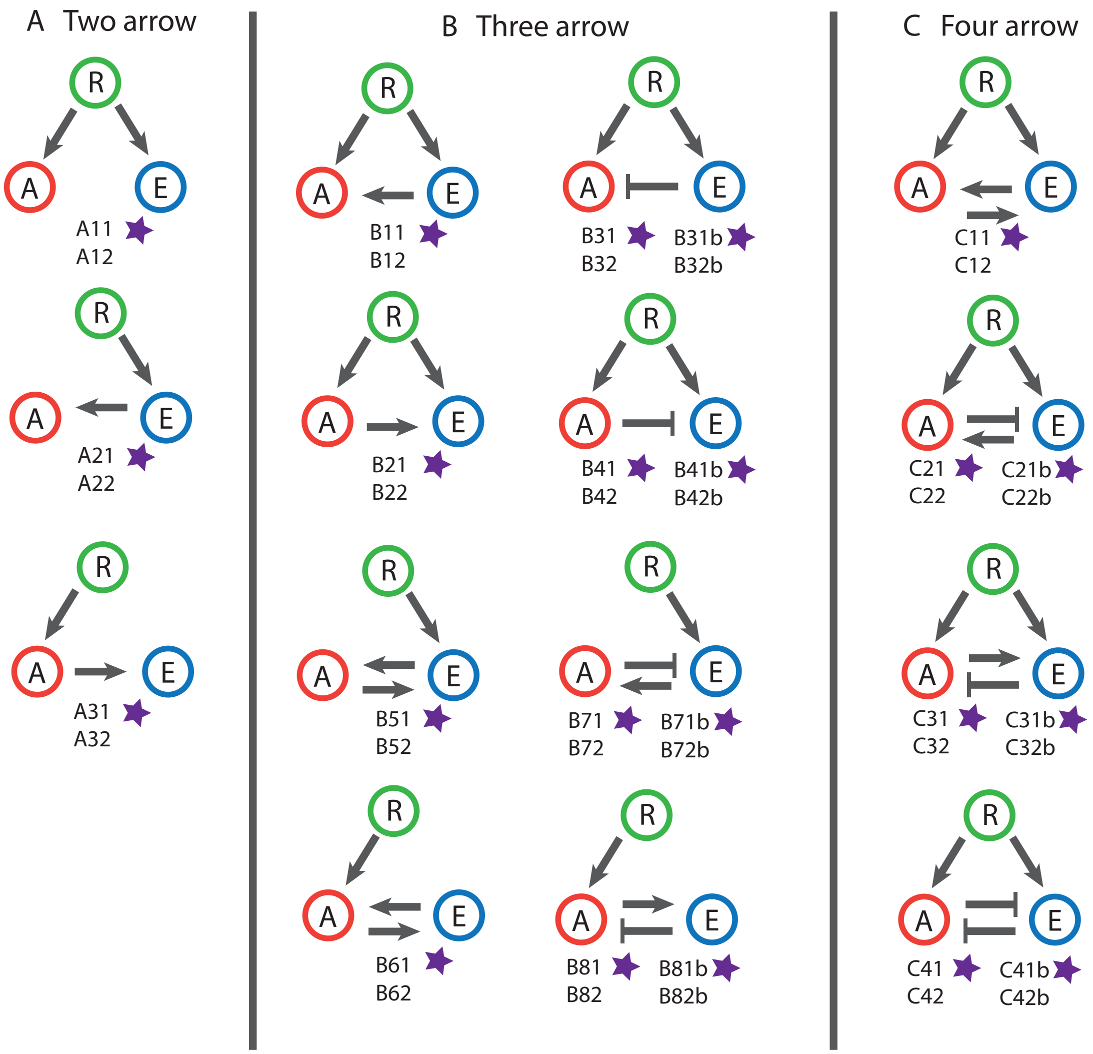

FIG. S8. Mechanistic models of breast cancer cell lines. The name of network model is indexed first by whether it is a two arrow (A), three arrow (B), or four arrow (C) model. The second index assigns a number to each network topology. Each network can be further subdivided to describe a model with mass action kinetics (1 in third index) and a model with Michaelis-Menten kinetics (2 in third index). Any network with inhibition (⊣) has 4 models considered: mass action removal (1 in third index), Michaelis-Menten removal (2 in third index), mass action blocking (1b in third index), or Michaelis-Menten removal (2b in third index). Stars next to the model name are globally structurally identifiable models, all other models are locally structurally identifiable.

are locally identifiability have a finite number of indistinguishable parameter values. Since we only have time-course measurements for A and E, we use a differential algebra approach for eliminating the species R. We test identifiability using the algorithm `DAISY` [20]. All of our models are locally, if not globally identifiable given the experimental data. Globally identifiable models are denoted by brown boxes in Fig. S8.

3. Parameter estimation

We estimate parameters using the average time-course for each cluster. The model simulated at a parameter vector $\boldsymbol{\theta}$ gives a vector of model predictions of E and A at dose L and time point t_j and data $\mathcal{D}$ is the set of normalised

Ligand	Associated Receptors
BTC	ErbB1, ErbB4
EGF	ErbB1
EPR	ErbB1, ErbB4
FGF-1	FGFR-1, FGFR-2, FGFR-3, FGFR-4
FGF-2	FGFR-1, FGFR-2, FGFR-3, FGFR-4
HGF	cMET
HRG	ErbB4
IGF-1	IGF1R
IGF-2	IGF1R, IGF2R
INS	InsR
NGF-beta	TrkA
PDGF-BB	PDGFRa, PDGFRb
SCF	c-Kit
VEGF165	VEGFR-1, VEGFR-2, VEGFR-3

TABLE IV. Receptors associated to each ligand

measurements of $\hat{E}$ and $\hat{A}$. The squared sum of errors of the model with parameter set $\boldsymbol{\theta}$ is:

$$E_{\mathcal{D}}(\boldsymbol{\theta}) = \sum_{L \in \text{ doses}} \sum_{t_j} \left(\hat{A}_{ij}(t_j;\, L) - A_{ij}(t_j;\, \boldsymbol{\theta},\, L)\right)^2 + \left(\hat{E}_{ij}(t_j;\, L) - E_{ij}(t_j;\, \boldsymbol{\theta},\, L)\right)^2.$$

We seek the parameter set $\boldsymbol{\theta}^*$ that minimises the discrepancy between the model and the data:

$$\boldsymbol{\theta}^* = \operatorname*{argmin}_{\boldsymbol{\theta}} E_{\mathcal{D}}(\boldsymbol{\theta}), \quad \text{subject to } \boldsymbol{\theta}^* \geq \mathbf{0}. \tag{D1}$$

We find $\boldsymbol{\theta}^*$ using the Squeeze-and-Breathe (SB) evolutionary optimisation algorithm [21], available as code here `https://people.maths.ox.ac.uk/beguerisse/#squeezecode`. Given an initial estimate of the distribution of the parameter values (a 'prior'). SB generates a large number of parameter sets using Monte-Carlo simulation. These points are used as the starting guess for a local minimisation of $E_{\mathcal{D}}(\boldsymbol{\theta})$ using a derivative free method such as Nelder-Mead [22]. The local minima are ranked and the best points are used to recompute the distribution of the parameters (the 'posterior'). These new posteriors are used as priors in a new iteration of the algorithm, which again looks for new local minima and keeps the best. This process goes on until the error function converges. One of the key advantages of SB is that it can handle situations where little is known about the parameter values by efficiently exploring the parameter space, even venturing to regions outside the original prior (for this reason the posteriors are not true posteriors in the Bayesian sense). Figure S9 shows an example for the type of output produced by SB.

4. Model selection

We perform model selection on the 40 models and 5 clusters using the Akaike Information Criterion with a correction for finite sample size (AICc) [23]. For a given model i with p_i parameters,

$$AICc_i = n \ln(nRSS_i) + 2p_i + \frac{2p_i(p_i+1)}{n-(p_i+1)},$$

where n is the number of observations and RSS_i is the residual sum of squares of the model. The AICc balances how well the model fits the data with the complexity of the model (the number of parameter values). The lowest AICc_i is the preferable model. Figure S10 shows the AIC scores of the models for each cluster, ranked from first (best) to tenth.

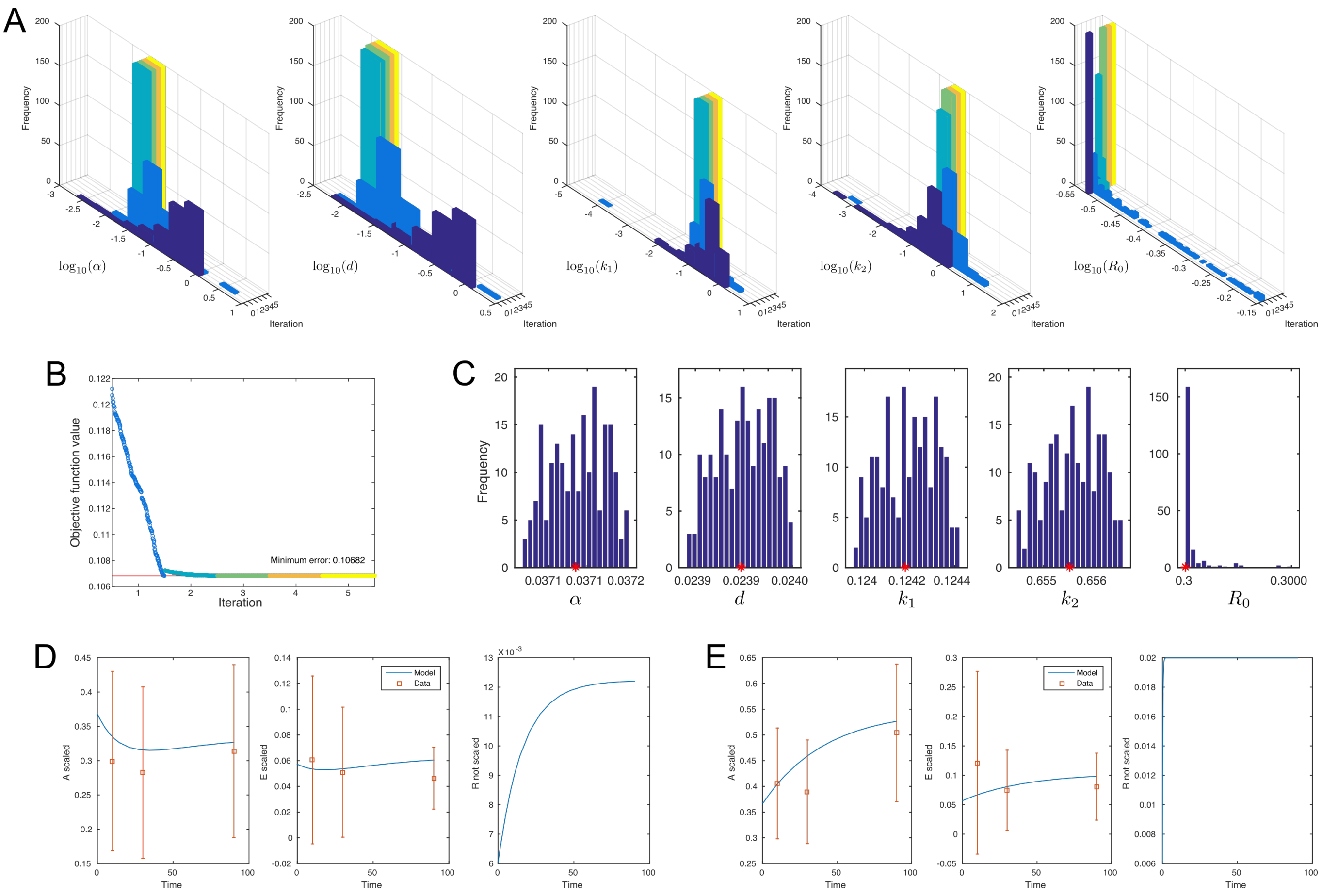

FIG. S9. Example output of the Squeeze and Breathe parameter estimation algorithm. A: Sequence of posteriors for each parameter after each iteration. B: Convergence of the objective function. The plot shows the error of best parameter sets after each iteration. C: Final distribution of the parameters after convergence. The red star marks the value of the parameter with the lowest error. D and E: Time course of the model with the best parameters for one cluster.

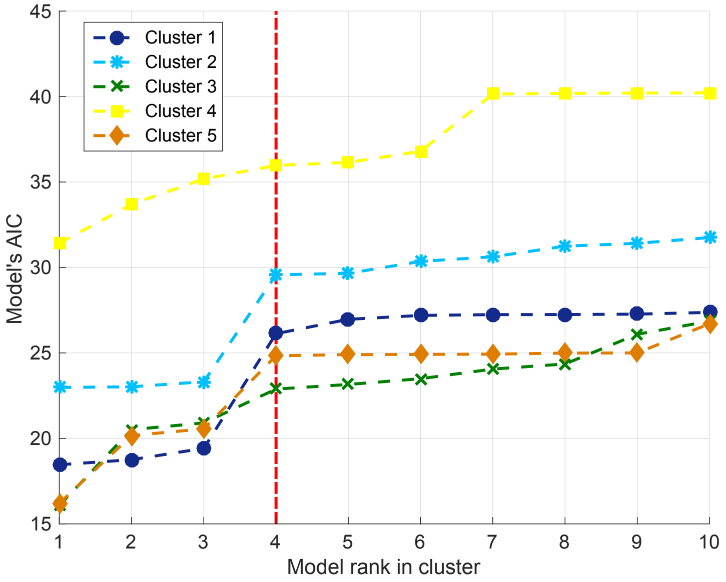

FIG. S10. Values of the AIC for the models ranked first to tenth in each of the five clusers depicted in Fig. 4 of the Main Text. The dashed red line indicates the cutoff for the models in Fig. 4.

[1] M. Niepel, M. Hafner, E. A. Pace, M. Chung, D. H. Chai, L. Zhou, J. L. Muhlich, B. Schoeberl, and P. K. Sorger, BMC Biol **12**, 20 (2014).
[2] B. Vangelov, *Unravelling Biological Processes using Graph Theoretical Algorithms and Probabilistic Models*, Ph.D. thesis, Imperial College London (2014).
[3] M. Beguerisse-Díaz, B. Vangelov, and M. Barahona, 2013 IEEE Global Conference on Signal and Information Processing (GlobalSIP) , 937 (2013).
[4] M. Beguerisse-Díaz, G. Garduño Hernández, B. Vangelov, S. N. Yaliraki, and M. Barahona, J R Soc Interface **11** (2014), 10.1098/rsif.2014.0940.
[5] J.-C. Delvenne, S. Yaliraki, and M. Barahona, Proc Nat Acad Sci USA **107**, 12755 (2010), http://www.pnas.org/content/107/29/12755.full.pdf+html.
[6] J.-C. Delvenne, M. T. Schaub, S. N. Yaliraki, and M. Barahona, in *Dynamics On and Of Complex Networks, Volume 2*, Modeling and Simulation in Science, Engineering and Technology, edited by A. Mukherjee, M. Choudhury, F. Peruani, N. Ganguly, and B. Mitra (Springer New York, 2013) pp. 221–242.
[7] M. Meilă, Journal of Multivariate Analysis **98**, 873 (2007).
[8] R. Heinrich, B. G. Neel, and T. A. Rapoport, Molecular Cell **9**, 957 (2002).
[9] C. Y. Huang and J. E. Ferrell, Proc Natl Acad Sci USA **93**, 10078 (1996), http://www.pnas.org/content/93/19/10078.full.pdf+html.
[10] A. von Kriegsheim, D. Baiocchi, M. Birtwistle, D. Sumpton, W. Bienvenut, N. Morrice, K. Yamada, A. Lamond, G. Kalna, R. Orton, D. Gilbert, and W. Kolch, Nature **11**, 1458 (2009).
[11] C. J. Marshall, Cell , 179 (1995).
[12] J. E. Purvis and G. Lahav, Cell **152**, 945 (2013).
[13] W. W. Chen, B. Schoeberl, P. J. Jasper, M. Niepel, U. B. Nielsen, D. A. Lauffenburger, and P. K. Sorger, Molecular Systems Biology **5** (2009), 10.1038/msb.2008.74, http://msb.embopress.org/content/5/1/239.full.pdf.
[14] J. Liepe, S. Filippi, M. Komorowski, and M. P. H. Stumpf, PLoS Comput Biol **9**, e1002888 (2013).
[15] K. A. Fujita, Y. Toyoshima, S. Uda, Y.-i. Ozaki, H. Kubota, and S. Kuroda, Sci Signal **3**, ra56 (2010).
[16] J.-K. Won, H. W. Yang, S.-Y. Shin, J. H. Lee, W. D. Heo, and K.-H. Cho, Journal of Molecular Cell Biology **4**, 153 (2012).
[17] D. Fey, D. R. Croucher, W. Kolch, and B. N. Kholodenko, Frontiers in physiology **3**, 355 (2012).
[18] M. Beguerisse-Díaz, R. Desikan, and M. Barahona, Journal of The Royal Society Interface **13** (2016), 10.1098/rsif.2016.0409, http://rsif.royalsocietypublishing.org/content/13/121/20160409.full.pdf.
[19] M. Niepel, M. Hafner, E. A. Pace, M. Chung, D. H. Chai, L. Zhou, B. Schoeberl, and P. K. Sorger, Sci Signal **6**, ra84 (2013).
[20] G. Bellu, M. P. Saccomani, S. Audoly, and L. D'Angiò, Computer Methods and Programs in Biomedicine **88**, 52 (2007).
[21] M. Beguerisse-Díaz, B. Wang, R. Desikan, and M. Barahona, J R Soc Interface **9**, 1925 (2012), http://rsif.royalsocietypublishing.org/content/9/73/1925.full.pdf+html.
[22] J. A. Nelder and R. Mead, The computer journal **7**, 308 (1965).
[23] K. Burnham and D. Anderson, *Model Selection and Multimodel Inference: A Practical Information-Theoretic Approach* (Springer New York, 2003).
